# *NTRK* fusions in osteosarcoma are rare and non‐functional events

**DOI:** 10.1002/cjp2.158

**Published:** 2020-02-05

**Authors:** Baptiste Ameline, Karim H Saba, Michal Kovac, Linda Magnusson, Olaf Witt, Stefan Bielack, Michaela Nathrath, Karolin H Nord, Daniel Baumhoer

**Affiliations:** ^1^ Bone Tumour Reference Center at the Institute of Pathology University and University Hospital Basel Basel Switzerland; ^2^ Department of Laboratory Medicine, Division of Clinical Genetics Lund University Lund Sweden; ^3^ Coordinator INFORM Program, Hopp Children's Cancer Center, German Cancer Research Center, University Hospital Heidelberg Heidelberg Germany; ^4^ Cooperative Osteosarcoma Study Group, Stuttgart Cancer Center, Klinikum Stuttgart – Olgahospital, Pediatrics 5 (Oncology, Hematology, Immunology) Stuttgart Germany; ^5^ Department of Pediatrics, Pediatric Oncology Center Technische Universität München Munich Germany; ^6^ Pediatric Hematology and Oncology Klinikum Kassel Kassel Germany

**Keywords:** NTRK, tyrosine kinase inhibitors, osteosarcoma

## Abstract

Neurotrophic tyrosine receptor kinase (*NTRK*) fusions are promising molecular targets that have been described in a broad range of malignant tumours. Fusions commonly lead to the expression of chimeric proteins with constitutive tyrosine kinase activation that drives tumorigenesis. Despite a low prevalence among most solid tumours (<1%), the first encouraging results with pan‐NTRK tyrosine kinase inhibitors (TKIs) such as larotrectinib or entrectinib stimulated the search for eligible patients. Here, we report the first three cases of osteosarcoma harbouring *NTRK* fusions, among 113 patients sequenced. It is also the first report on *NTRK* fusions within a tumour type characterised by highly rearranged genomes and abundant passenger mutations. Whereas the presence of *NTRK* gene fusions in many tumours is considered to be one of the main driver events for tumour progression, the three chimeric transcripts described here appear non‐functional and likely represent randomly occurring passenger alterations. Particularly in tumours with complex karyotypes, it may therefore be advisable to specifically investigate the fusion transcripts for functional impact before considering targeted treatment approaches using pan‐NTRK TKIs.

## Introduction

Physiologically expressed in neuronal tissue, the *NTRK1*, *NTRK2* and *NTRK3* genes promote proliferation and survival of neuronal cells through the activation of the MAP‐kinase, PLC‐γ and PI3K‐AKT signalling pathways 1. Gene fusions between the tyrosine kinase domain of *NTRK* genes and different upstream partners lead to ectopic expression of constitutively active chimeric proteins. Among the numerous fusion partners already described, most are activating translocations harbouring dimerisation domains responsible for the tyrosine kinase overactivation 1. The list of cancer types in which *NTRK* fusions have been identified has kept growing since their discovery in 1982 2. These tumours can be divided into a group of rare malignancies displaying a high prevalence (>80%) and a group of various other cancer types, in which *NTRK* fusions are generally infrequent (<5%) 3.

*NTRK* fusions are observed in a variety of configurations differing in the combination of N‐terminal partners, the *NTRK* gene involved, the downstream pathways activated and the tumour types affected. Nevertheless, the pan‐NTRK TKI larotrectinib has shown remarkable efficacy independent of tumour type with an overall response rate > 75% 4. Similarly, entrectinib, a Pan‐NTRK/ROS1/ALK inhibitor displayed an objective response rate of 79% over different solid tumour types 5, 6.

Our aim was to search for *NTRK* fusions in a comprehensive set of 113 osteosarcomas. Since 30–40% of patients with osteosarcoma still die of their disease despite intense and multimodal treatment regimens, innovative and treatable targets are urgently needed.

## Material and methods

### Sample collection

All tumour samples were re‐evaluated by an experienced bone pathologist and confirmed the diagnosis of conventional high‐grade osteosarcoma and a tumour content of >50% per sample. Ethical approval was given by the Ethikkommission beider Basel (reference 274/12) and by the Regional Ethics Committee of Lund University.

### DNA sequencing for the detection of structural aberrations

The DNA sequencing strategy differed slightly for the samples from Basel and Lund. In Basel, paired‐end libraries from tumour and paired‐blood DNA were prepared using the Agilent SureSelectXT HumanV5 kit for whole‐genome sequencing (WGS). These were sequenced together with a tumour complementary DNA on an Illumina HiSeq2500 (Cambridge, UK) (paired‐end 100 bp). Sequencing reads were mapped to the GRCh37 human reference genome using BWA as described before 7. In Lund, DNA was extracted form fresh‐frozen tumour biopsies and mate pair libraries were prepared for sequencing using the Nextera mate pair sample preparation kit (Illumina, Cambridge, UK) as previously described 8. To identify structural rearrangements, the sequence data were analysed using the structural variant callers TIDDIT and Delly2.

### Circos plots

Copy number aberrations were detected by segmenting log2 values extracted from SNP array analyses using the R package ‘copynumber’. For WGS, copy number segments were generated with ‘cnvkit’ using matched normal tissue as a baseline for copy number = 2. Copy number and structural variant data were then combined to construct circos plots using the R package ‘RCircos’.

### RNA sequencing

RNA sequencing in Lund was performed as described previously 8. In Basel, sequencing libraries were prepared using the TruSeq RNA Sample Preparation Kit v2 (Illumina). Total RNA was extracted from fresh‐frozen tumour tissue and mRNA was then purified from 1 μg of total RNA using oligo(dT) beads. Paired‐end sequencing was performed on the Illumina HiSeq 2500 in rapid run mode according to the manufacturer's protocol using the TruSeq SBS Kit v3. Sequencing reads were mapped to the GRCh37 human reference genome using STAR or Hisat2.

### Fusion transcript detection

ChimeraScan, deFuse, and FusionCatcher algorithms were used to detect chimeric transcripts from RNA‐seq fastq files. Predicted fusions were filtered out based on the presence of chimeric spanning or encompassing reads. The sequences of reads spanning a *NTRK* gene were then blasted against the human transcriptome in order to exclude any ambiguity concerning the involved partners.

### RT‐PCR and Sanger sequencing validation

RT‐PCR and Sanger sequencing were carried out as described previously 8. In brief, the remaining mRNAs from two patients (*VPS18‐NTRK3*; *RALGPS2‐NTRK3*) were retrotranscribed into cDNAs. RT‐PCR was performed with paired primers designed within 200 bp around the breakpoint. The amplification products were then Sanger sequenced.

### Immunohistochemistry

IHC staining was performed using a pan‐Trk monoclonal antibody (clone EPR17341, Abcam, Cambridge, UK) as described elsewhere 9.

## Results

**Figure 1:**
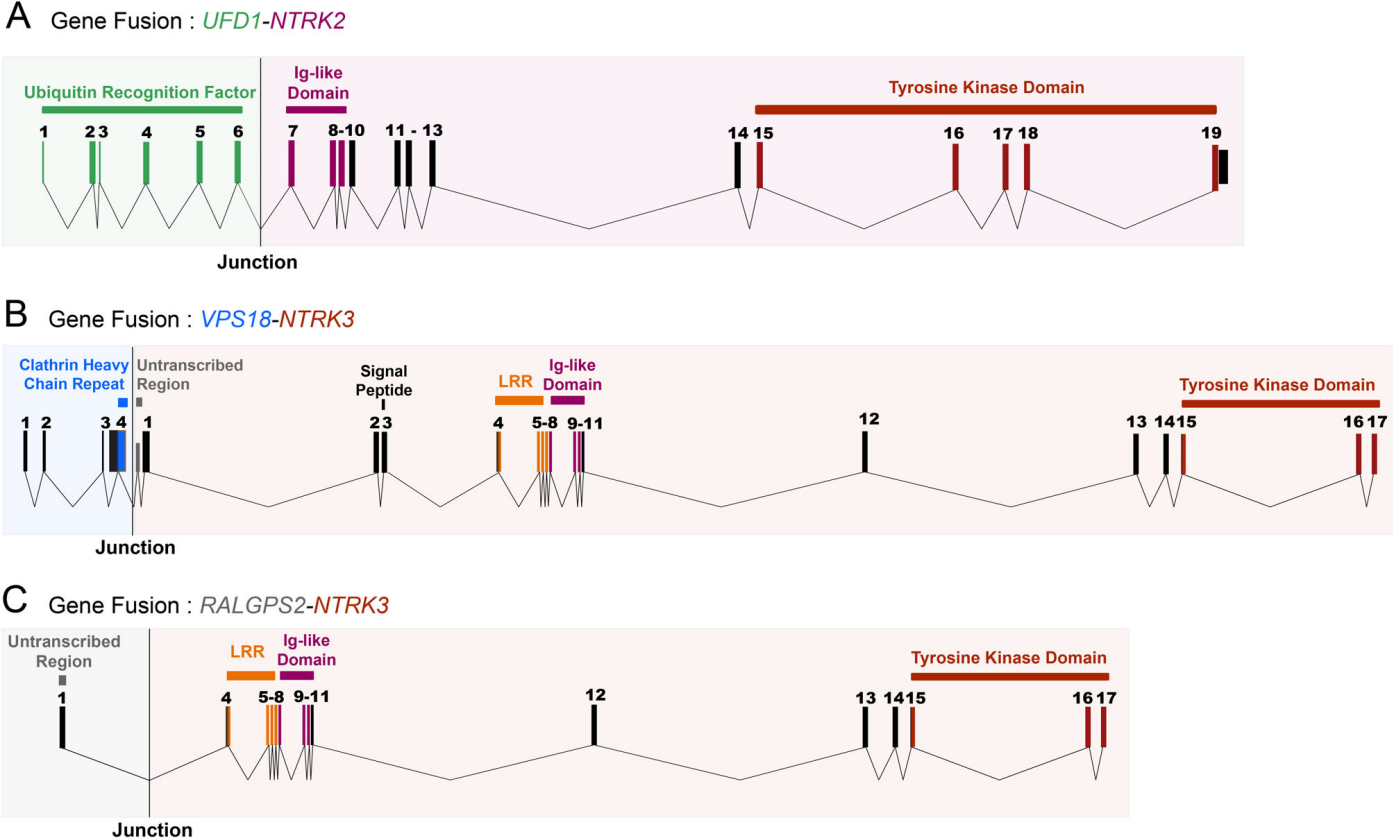
Schematic representation of the *NTRK* gene fusions found in patients with osteosarcoma. (A) Rearrangement between introns 6 of both *UFD1* and *NTRK2* genes. (B) Gene fusion occured between intron 4 of the *VPS18* gene and the 5′ untranslated region of the *NTRK3* gene. (C) Rearrangement between the 5′ unstranslated region of the *RALGPS2* gene and intron 3 of the *NTRK3* gene.

**Figure 2:**
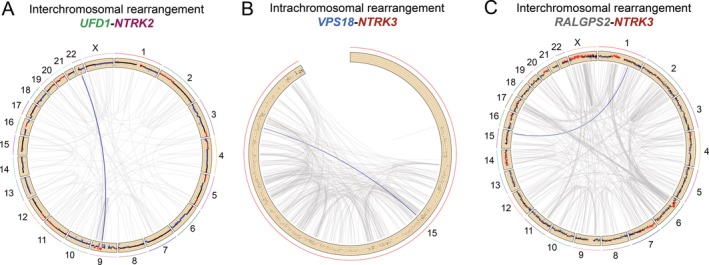
Circos plots displaying the NTRK gene fusions among numerous structural variants in highly rearranged genomes. (A) Rearrangements between chromosomes 9 and 22 result in a *UFD1‐NTRK2* fusion in Case 1. (B) Chromothripsis for chromosome 15 results in a *VPS18‐NTRK3* fusion in Case 2. (C) Rearrangements between chromosomes 1 and 15 result in a *RALGPS2‐NTRK3* fusion in Case 3. Blue lines represent the *NTRK* rearrangements and grey lines represent other structural variations. Genomic copy numbers are displayed in the first track. The dots represent the log2 values for normal (black), lost (blue) and gained (red) regions.

Next‐generation DNA and RNA sequencing was performed across 113 osteosarcomas, including samples from primary tumours and metastases (76 and 37 cases, respectively). Assessment and comprehensive analyses of chimeric transcripts were carried out using ChimeraScan 10, FusionCatcher 11 and defuse 12 and resulted in the detection of *NTRK* fusions in three patients (2.7% of cases, *n* = 113; Figure 1 and see supplementary material, Figure S1). All gene fusions were verified and validated by the existence of split‐reads in genome sequencing data (Figure 2) and/or RT‐PCR (see supplementary material, Figure S2). Somatic copy number variations and structural variant assessment derived from genome sequencing furthermore showed that the *NTRK* fusions occurred in the context of heavily recombined genomes (Figure 2).

**Figure 3:**
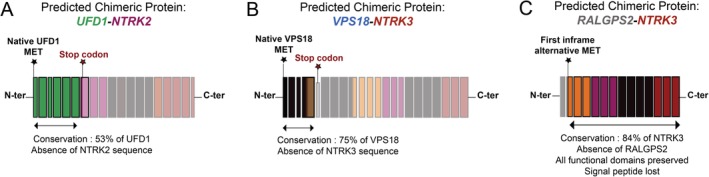
Schematic representation of NTRK fusion proteins according to their transcriptomic features. Representation of NTRK fusion proteins in cases with translation of chimeric transcripts. The faded colours represent the functional domains missing due to the recombination. (A) Fusion protein involving UFD1 and NTRK2. (B) Fusion protein between VPS18 and the 5′ untranslated region of the *NTRK3* gene. (C) Chimeric protein resulting from the gene fusion between the 5′ untranslated region of the *RALGPS2* gene and the *NTRK3* gene. In the absence of a start codon from the 5′ partner, only the protein sequence of NTRK3 could be translated.

In the first case, we analysed a 23‐year old female with lung metastases and identified a novel fusion of *NTRK2* with an upstream partner *UFD1*. The fusion led to a premature stop codon by introducing a reading frame shift in *NTRK2* upstream of the tyrosine kinase domain with subsequent shortening of the coding sequence of the *NTRK2* transcript (Figure 3A).

**Figure 4:**
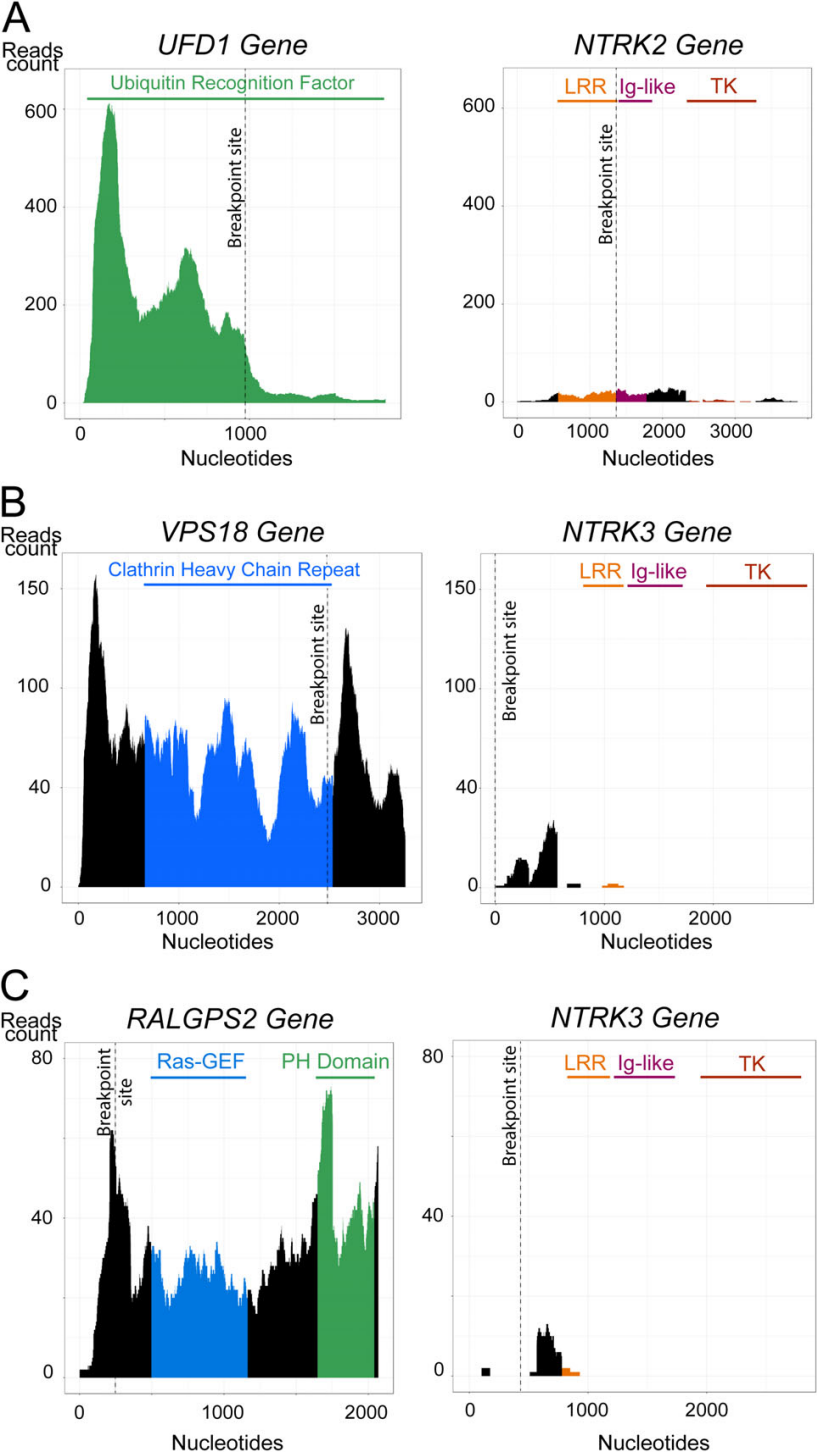
Reads count for each partner of *NTRK* gene fusions. Number of RNA sequence reads at each nucleotide position. The dashed lines represent the junction site with the fusion partner. The colour code highlights the nucleotide sequences coding for the functional domains. Abbreviations: LRR, leucine‐rich reapeat; TK, tyrosine kinase.

The second analysis of a locally recurring osteosarcoma of a 22‐year old female revealed an intra‐chromosomal rearrangement between *NTRK3* and the *VPS18* gene that was likely generated through chromothripsis of chromosome 15 (Figure 2B). No fusion transcripts were fully transcribed (Figure 4B). Moreover, the rearrangement led to the introduction of a premature stop codon, preventing any translation of NTRK3 functional domains (Figure 3B). A *VPS18*‐*NTRK3* gene fusion has already previously been reported by Okamura *et al* in an unspecified tumour type, although no details about the translocation breakpoint were provided 6.

The third osteosarcoma was derived from a local recurrence of a 51‐year old female and showed a dicentric translocation between the 5′ untranslated region of the *RALGPS2* gene and exons 4–17 of *NTRK3*. The predicted fusion transcript lacked an endogenous start codon (Figure 1C) and, although it introduced an alternative in‐frame start codon just after the breakpoint, this was not sufficient to induce transcription of the downstream functional domains of *NTRK3* (Figures 3C and 4C).

Immuohistochemistry was performed using a pan‐Trk antibody and did not yield immunoreactivity in any of the three osteosarcomas with *NTRK* rearrangements (data not shown), indicating a lack of detectable protein.

All tumour samples with *NTRK* fusions had been obtained and archived >3 years before this study was conducted so none of the patients has been considered for pan‐NTRK TKI treatment.

Regarding additional genetic alterations observed, the three tumours all showed copy number losses of *CDKN2A* and *CDKN2B*. This concomitant deletion has already been reported in several *NTRK*‐fusion positive tumours of different entities 13, 14.

## Discussion

To the best of our knowledge, this is the first study to describe *NTRK* gene fusions in osteosarcoma. A previous pan‐cancer study did not reveal a single *NTRK* fusion in the 53 osteosarcomas included 6. As expected, osteosarcoma does not belong to the group of tumours that show a high prevalence of *NTRK* fusions as observed in a small set of rare neoplasms including secretory carcinoma of the breast / salivary gland or infantile fibrosarcoma 3, 6.

*NTRK* gene fusions are commonly considered oncogenic drivers regardless of tumour type but chromosomally unstable tumours like osteosarcomas might challenge this notion. The high amount of chromosomal instability increases the likelihood of abundant and randomly occurring passenger alterations that might also involve the *NTRK* genes. Whether individual *NTRK* gene fusions actually represent driver events or rather non‐functional epiphenomena, however, seems crucial when targeted treatment approaches are considered.

The studies published so far included patients with evidence of *NTRK* rearrangements based on immunohistochemistry (IHC), FISH or RNA/DNA sequencing methods, and some studies only required a tumour to be ‘positive for a molecular alteration’ of *NTRK1‐3* 4, 5 (http://clinicaltrials.gov number: NCT02097810, NCT02568267, NCT02122913, NCT02576431 and NCT02637687). Recent studies suggesting that IHC with a pan‐NTRK antibody reliably identifies *NTRK1‐3* rearrangements pave the way for rendering individual patients suitable for pan‐NTRK TKI therapy based solely on surrogate markers 9. Accordingly, the three patients with *NTRK* fusions described here would have met the criteria for enrollment in these studies. However, DNA and RNA sequencing of the two first patients demonstrate the introduction of premature stop codons by these fusions and a lack of transcription (Figure 4), which is tantamount to a loss‐of‐function of the chimeric proteins due to the absence of tyrosine kinase domains. Frameshifts in *NTRK* fusion transcripts have been reported in only a single case of a primary undifferentiated neuroendocrine carcinoma so far 15. Finally, the third case exemplifies a recombined transcript whose functionality cannot be assessed by our analysis, although the absence of both an endogenous start codon and detectable RNA makes subsequent translation highly unlikely. None of the three tumours had detectable expression of NTRK proteins, as expected in the absence of transcription.

In summary, the role of *NTRK* gene fusions as driving/oncogenic events in the three osteosarcoma cases described here can be virtually excluded. At the same time, the functionality of *NTRK* chimeric transcripts detected in other tumour types with highly rearranged genomes should be interpreted with caution. Chromoanagenesis could be a potential mechanism to explain non‐functional *NTRK* gene fusions as observed in the second tumour. As long as FISH or any other breakpoint‐independent technique alone are used as inclusion criteria for clinical trials investigating pan‐NTRK TKIs, patients will be included who will most probably not respond to treatment. Hence, sequencing of the fusion transcripts in at least all highly rearranged tumours, but preferably in all tumours, should be considered before initiating targeted treatment.

## Author contributions statement

DB, BA and MK conceived and designed the study. BA, KHS, LM and KHN acquired, analysed or interpreted data. BA and DB drafted the manuscript. KHN, SB, KHS, NN and MK critically revised the manuscript for important intellectual content. LM, SB, MN and OW provided administrative, technical or material support. DB supervised the study.

## Supporting information


**Figure S1.** Chimeric reads spanning the breakpoint site
**Figure S2.** Sanger sequencing after RT‐PCR amplication of the *RALGPS2‐NTRK3* fusion transcript
