# Supplementary material for: NTRK fusions in osteosarcoma are rare and non‐functional events
Source: J Pathol Clin Res. 2020 Feb 5;6(2):107–12. doi: 10.1002/cjp2.158 (PMC7164374; doi:10.1002/cjp2.158)
Supplement: Supplementary file 1 — Figure S1. Chimeric reads spanning the breakpoint site Figure S2. Sanger sequencing after RT‐PCR amplication of the RALGPS2‐NTRK3 fusion transcript [file CJP2-6-107-s001.docx]

***NTRK* fusions in osteosarcoma are rare and non-functional events**

Ameline B *et al*. *J Pathol Clin Res* DOI: 10.1002/cjp2.158

**Supplementary Figures**


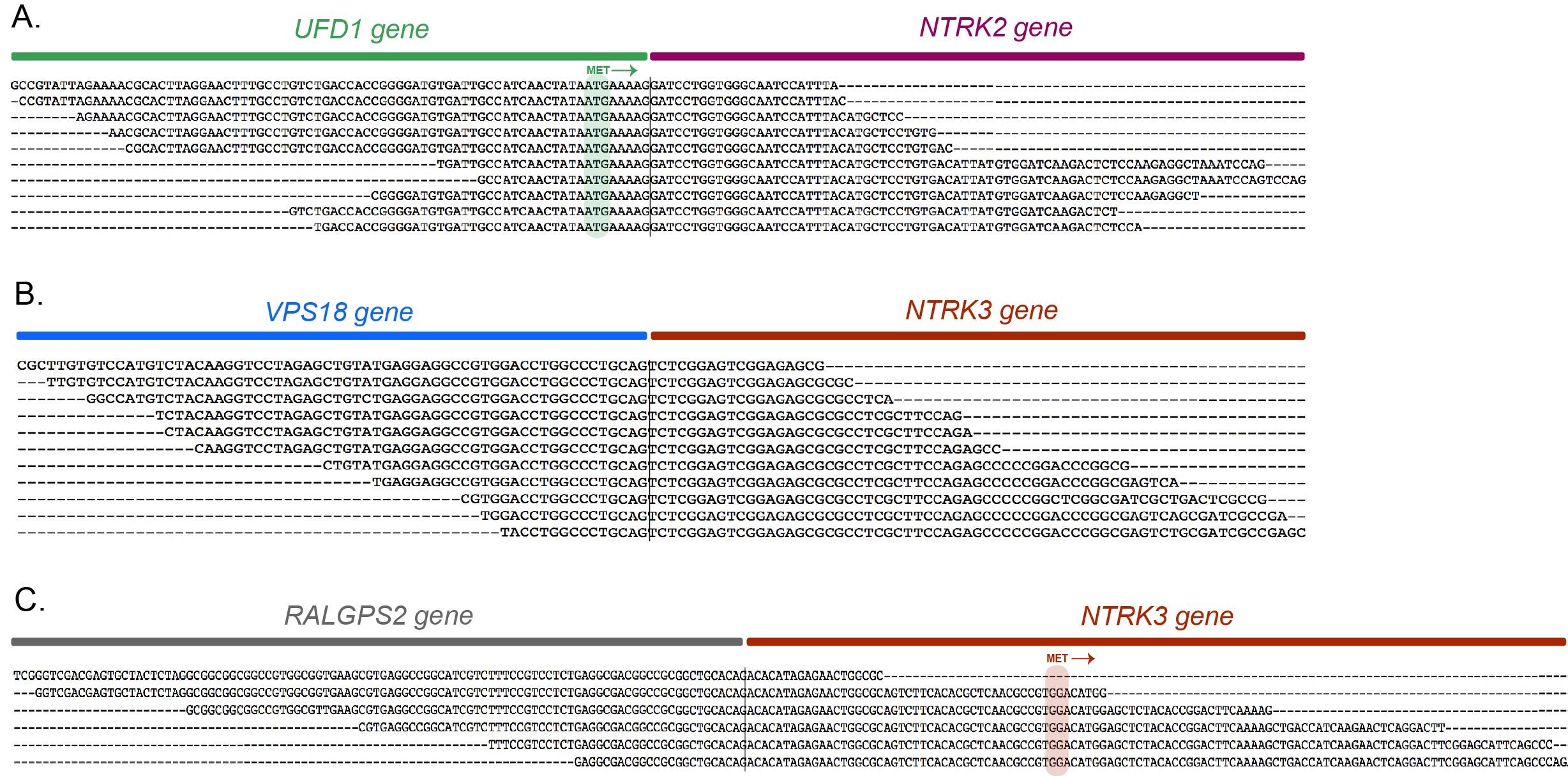


## **Figure S1.** Chimeric reads spanning the breakpoint site. Each unique RNA reads detected with either ChimeraScan or FusionCatcher as supporting spanning reads of NTRK gene fusions were aligned using Clustal Omega.


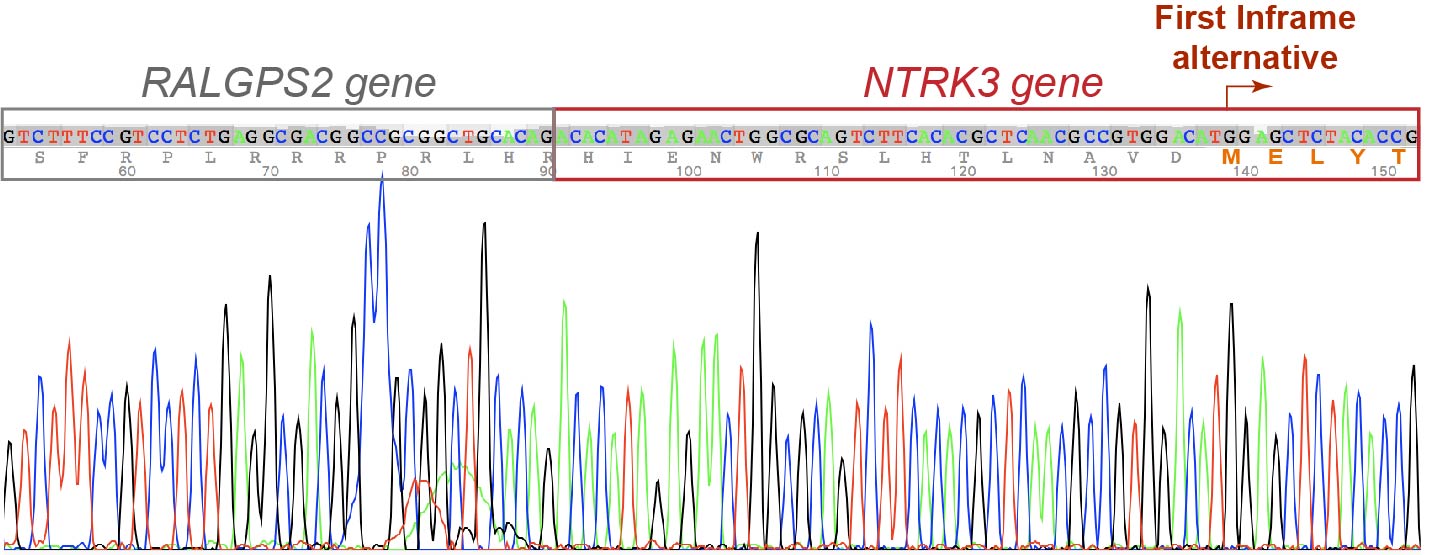


## **Figure S2.** Sanger sequencing after RT-PCR amplication of the *RALGPS2-NTRK3* fusion transcript.
